# Genetic Influence on Nociceptive Processing in the Human Brain—A Twin Study

**DOI:** 10.1093/cercor/bhab206

**Published:** 2021-07-21

**Authors:** Gránit Kastrati, Jörgen Rosén, William H Thompson, Xu Chen, Henrik Larsson, Thomas E Nichols, Irene Tracey, Peter Fransson, Fredrik Åhs, Karin B Jensen

**Affiliations:** Department of Clinical Neuroscience, Karolinska Institutet, SE-171 77, Stockholm, Sweden; Department of Psychology and Social Work, Mid Sweden University, SE-831 25, Östersund, Sweden; Department of Psychology and Social Work, Mid Sweden University, SE-831 25, Östersund, Sweden; Department of Clinical Neuroscience, Karolinska Institutet, SE-171 77, Stockholm, Sweden; Department of Biomedical Data Sciences, Leiden University Medical Center, 2300 RA, Leiden, the Netherlands; Department of Medical Sciences, Örebro University, SE--701 82, Örebro, Sweden; Oxford Big Data Institute, Li Ka Shing Centre for Health Information and Discovery, Nuffield Department of Population Health, University of Oxford, OX3 7LF, Oxford, UK; Wellcome Centre for Integrative Neuroimaging, Nuffield Department of Clinical Neurosciences, University of Oxford, OX3 9DU, Oxford, UK; Department of Clinical Neuroscience, Karolinska Institutet, SE-171 77, Stockholm, Sweden; Department of Psychology and Social Work, Mid Sweden University, SE-831 25, Östersund, Sweden; Department of Clinical Neuroscience, Karolinska Institutet, SE-171 77, Stockholm, Sweden

**Keywords:** pain, nociception, genetics, functional connectivity, fMRI

## Abstract

Nociceptive processing in the human brain is complex and involves several brain structures and varies across individuals. Determining the structures that contribute to interindividual differences in nociceptive processing is likely to improve our understanding of why some individuals feel more pain than others. Here, we found specific parts of the cerebral response to nociception that are under genetic influence by employing a classic twin-design. We found genetic influences on nociceptive processing in the midcingulate cortex and bilateral posterior insula. In addition to brain activations, we found genetic contributions to large-scale functional connectivity (FC) during nociceptive processing. We conclude that additive genetics influence specific brain regions involved in nociceptive processing. The genetic influence on FC during nociceptive processing is not limited to core nociceptive brain regions, such as the dorsal posterior insula and somatosensory areas, but also involves cognitive and affective brain circuitry. These findings improve our understanding of human pain perception and increases chances to find new treatments for clinical pain.

## Introduction

Nociceptive processing is crucial for survival as it provides an organism with information about potential or actual tissue damage. The neural processes underlying this capacity are evolutionary conserved, as evolved nociceptive systems are observed in a variety of species (Walters and Williams 2019). In humans, neuroimaging studies have established a large network of brain regions that consistently activate in response to nociceptive information (Jensen et al. 2016). Most such activations are evoked independently of type of nociceptive input and can be found in infants with minimal prior exposure to pain (Goksan et al. 2015). This suggests that genes modulate basic aspects of nociceptive processing in the human brain.

There is considerable variation in nociception between individuals and attempts have been made to determine the genetic influence on such differences (Mogil 2012). The genetic influence on sensitivity to experimental pain, for example, has been investigated by comparing identical and fraternal twins and estimated to 26% for heat- and 60% for cold-induced pain (Nielsen et al. 2008). Another study found similar genetic influence on individual sensitivity to pain, ranging from 22% to 55% depending on pain modality (Norbury et al. 2007). Studies that link single-nucleotide polymorphisms to functional neuroimaging data (e.g., Zubieta et al. 2003; Oertel et al. 2008; Vachon-Presseau et al. 2016) and studies of rare genetic mutations that affect pain perception (Salomons et al. 2016) suggest that our genes influence nociceptive processing, and our subjective experience of pain. Yet, the specific neural mechanisms and magnitude of such influence needs to be determined.

The experience of pain involves cross-communication between both nociceptive and non-nociceptive brain regions (Kucyi and Davis 2015; Geuter et al. 2020). To capture genetic influences on nociceptive processing, it is therefore relevant to move beyond mere activations in specific brain regions, to consider also their interactions. Recent advances in the neurosciences has seen a rapid increase in studies that model the brain as a large-scale network, which allows for estimating the degree of the interaction or cross-communication between brain regions and/or subnetworks (Bullmore and Bassett 2011; Sporns 2013). For example, the default-mode network that consistently activates during rest and deactivates when engaged in a task, show increased deactivation during painful tasks (Kong et al. 2010; Kucyi et al. 2013). Recent findings also show decreased functional connectivity (FC) between the primary somatosensory cortex and the default-mode network in chronic low back pain (Kim et al. 2019). Several studies have elucidated the relationship between genetics and functional brain network topology by means of functional Magnetic Resonance Imaging (fMRI), both for resting-state (Glahn et al. 2010; Fornito et al. 2011; Xu et al. 2017; Miranda-Dominguez et al. 2018; Reineberg et al. 2019) and experimental tasks (Alstott et al. 2009; Yang et al. 2016; Colclough et al. 2017). Estimates of the genetic influence of resting-state brain networks are replicable across studies and imply genetic influences on large-scale networks (Adhikari et al. 2018).

In this study, we estimated the genetic influence on nociceptive processing in the brain. A total of 246 twins (56 identical pairs; 67 fraternal pairs) participated in a fMRI study that included an aversive conditioning paradigm using electrical shocks. The aim was to estimate the genetic influence on 1) neural responses in pain processing regions and 2) whole-brain FC during nociceptive processing, as described in our preregistration protocol (https://osf.io/zesw5). To achieve our first aim, we constrained our analysis to pain processing regions defined independently of the current study (Wager et al. 2013). Regarding the second aim, we used a whole-brain parcellation scheme to study task-based FC.

## Materials and Methods

### Subjects

Twins between ages 20 and 60 years were recruited for the present study through the Swedish Twin Registry (STR). The STR contains > 194 000 twins and representsean epidemiological resource for the study of genetic and environmental influences on human traits, behaviors, and diseases (https://ki.se/en/research/the-swedish-twin-registry). Twin pairs with known zygosity were selected based on their capability to undergo magnetic resonance imaging as well as screened for substance abuse, ongoing psychological treatment or medicine affecting emotion or cognition. Only same-sex twin pairs were included in this study and after initial screening, 305 participants were recruited to the study and underwent fMRI scanning. Imaging data were excluded from the analysis if one of the following criteria were fulfilled 1) excessive amount of head motion (>50% of the data frames contained framewise displacement above 0.5 mm) (*n* = 16); 2) presence of outliers in terms of amplitude of brain responses. We here used the median absolute deviation method (Leys et al. 2013) to detect and outliers (here meaning a mean blood oxygenated level-dependent [BOLD] response deviating > 3 times the medial standard deviation across the whole sample). Imaging data from participants deemed to be outlies were removed together with data from their co-twin and not used in the subsequent analysis (*n* = 8); and 3) missing data/incomplete data collection from both twin pairs (*n* = 35). The final sample (*n* = 246) included 56 identical (35 female and 21 male) twin pairs (age: *M* = 34, SD = 8) and 67 fraternal (39 female and 28 male) twin pairs (age: *M* = 33, SD = 11). All participants provided written informed consent in accordance with the Uppsala Ethical Review Board Guidelines. Participants received reimbursement of SEK 1000 (roughly equal to 100 USD) for their participation.

### Brain Imaging

Imaging data were acquired using a 3.0 T scanner (Discovery MR750, GE Healthcare) and an 8-channel head-coil. Foam wedges, earplugs, and headphones were used to reduce head motion and scanner noise. We acquired T1-weighted structural images with whole-head coverage, time repetition (TR) = 2.4 s, time echo (TE) = 2.8 s, acquisition time 6.04 min and flip angle 11 (degrees). Functional images were acquired using gradient echo-planar-imaging (EPI), TR = 2.4 s, TE = 28 ms, and flip angle= 80 (degrees), with 47 seven volumes acquired with slice thickness 3.0 mm^3^ (no spacing, axial orientation, and phase-encoding direction A/P). The slices were acquired in an interleaved ascending order. Higher order shimming was performed, and 5 dummy scans were acquired before the experiment.

### Stimuli and Contexts

Visual stimuli were presented on a flat screen in the MR scanner via a projector (Epson EX5260) (see Supplementary Fig. S1). The computer running the stimulus presentation used a custom version of Unity (version 5.2.3, Unity Technologies) and communicated with BIOPAC for electrical stimuli (BIOPAC Systems) through a parallel port interface. The software for the parallel port interface was custom made and used standard .NET serial communication libraries by Microsoft (Microsoft Corporation).

### fMRI Paradigm Design

Noxious electrical stimuli were administered as part of a fear conditioning procedure. The paradigm was used to test genetic aspects of fear acquisition and results that focus on neural responses to trials that did not include an electrical shock will be reported elsewhere. Two virtual characters served as visual stimuli (CS) and were presented at a distance of 2.7-m projected on a screen in the MR scanner (see Supplementary Fig. S1). One of the virtual characters served as the aversive cue (CS+) and preceded the electrical stimuli whereas the other virtual character served as a safety cue (CS−). Stimuli serving as CS+ and CS− were counterbalanced across participants. Each of the cues appeared for 6 s. Participants were not told which character would be associated with electrical shocks. Prior to the conditioning phase, a habituation phase took place, during which each CS was presented 4 times without any electrical shocks. During conditioning, each cue type was displayed 16 times. Eight of the aversive cues co-terminated with presentation of the electrical shock (US) and 8 of the aversive cues did not include a shock. Four stimulus presentation orders were used to counterbalance the timing of CSs across subjects. An interstimulus interval (randomized jittering) followed each trial, with no cues present for 8–12 s. Total duration for the conditioning task was 9 min and 47 s. The initial 8 presentations (habituation) were not considered for this analysis.

The electrical shocks were delivered to the distal part of the participant’s left volar forearm (adjacent to the wrist) via radio-translucent disposable dry electrodes (EL509, BIOPAC Systems, Goleta, CA). As the present study also served to investigate fear acquisition, that is, neural responses to trials that did not include electrical stimulation (to be published elsewhere), the US presentation was brief (16 ms). Shock delivery was controlled using the STM100C module connected to the STM200 constant current stimulator (BIOPAC Systems), using a unipolar pulse with a fixed duration of 67 Hz. The physical voltage was individually calibrated before the experimental task using an ascending staircase of electrical currents until shocks were rated as “aversive” (Rosen et al. 2019). After finding the physical voltage that participants rated as aversive, this parameter was kept constant throughout the experiment. The determined average electrical voltage was *M* = 31 V, SD = 7, and range = (15, 55).

### Analysis of fMRI Imaging Data

Analyses of fMRI-data were performed using SPM12 (Welcome Department of Cognitive Neurology, University College, London, https://www.fil.ion.ucl.ac.uk/spm). Preprocessing of functional image volumes included interleaved slice time correction, realignment, co-registration to the T1-weighted image, spatial normalization to Montreal Neurological Institute (MNI) space (MNI152NLin6Asym), and spatially smoothed with an 8-mm Gaussian kernel.

In the first-level analysis, an event-related approach was used to estimate BOLD responses during nociceptive processing. Three event types were modeled, using separate regressors: The aversive cue that preceded the US (CS+_US_), the same CS+ that did not precede the US (CS+_no US_), and the electrical shock itself (US). Note that the aversive cue (CS+) co-terminated with the onset of the US 50% of the times. The duration of the visual cue (CS+) was set to 6 s and the US to 3 s. The first-level contrast for each participant that was latter used to estimate the genetic influence *h^2^* on nociceptive processing per se was modeled as (CS+_US_ & US > CS+_no US_). Since the aversive cue (CS+_US_) was immediately followed by the US, without any delay, the CS+_US_ and US were combined. The same visual cue (CS+_no US_), not followed by the US, was then subtracted in order to estimate the neural correlates to nociceptive processing per se. The group-level result for the same contrast is found in Supplementary Table S1 and Supplementary Figure S2. The statistical significance threshold was set to *P* < 0.05, family-wise error corrected (FWE) for multiple comparisons. Anatomical labeling of significantly activated brain regions were performed using the SPM Anatomy toolbox v.2.2c (Tzourio-Mazoyer et al. 2002).

### Defining the Functional Connectome in Response to Nociceptive Input

To investigate task-specific FC, the CONN FC toolbox was used (Whitfield-Gabrieli and Nieto-Castanon 2012) (http://www.nitrc.org/projects/conn, version 18b). As input to the CONN toolbox, we used the same preprocessing pipeline as outlined above except for removing the spatial smoothing. This decision was to minimize a spurious increase in local connectivity that would be induced otherwise. Subsequently, image data underwent ART-based outlier detection of volumes (version 2015-10) followed by image scrubbing. For the scrubbing procedure, we used a liberal threshold of the 99th percentile of normative sample, with a global-signal *z*-value threshold of 9 standard deviations and a subject motion threshold of 2 mm. Next, confounders were removed from the data. These consisted of the effect of each task (in order to remove constant task-induced responses in the BOLD signal), cerebrospinal fluid, white matter, SPM covariates (6 motion parameters and their quadratic effect), and regressors for scrubbing per individual (one regressor for each volume deemed a potential outlier; from zero to a maximum of 25 regressors per individual). Finally, image data were low-pass filtered [0.008 and 0.09]. BOLD time-series were extracted using a parcellation scheme with 400 nodes (Schaefer et al. 2018). We computed first-level weighted ROI-to-ROI functional connectivity (wFC) by computing task-specific bivariate correlation using weighted least squares (WLS), with weights defined as condition timeseries convolved with a canonical hemodynamic response function. Results were Fisher-transformed correlation coefficients between each pair of nodes. The first-level contrasts were modeled in the same way as described above for brain activations (CS+_US_ & US > CS+_no US_). Figure 2*A* and *C* shows the group-level result for the same contrast. For visualization purpose, we computed the within-network and between-network sum of FC between each pair of networks (Fig. 2*C*). For each network, say *A* and *B*, we sum the FC between *A* and *B* and divide by the number of nodes contained in the 2 networks. If *A = B,* the result is the sum of the within-network connectivity; otherwise the result is the between-network connectivity.

### Estimation of Genetic Influences on Brain Function

*Exclusion of outliers:* We identified univariate outliers in our data sample using the median absolute deviation method (Leys et al. 2013). Any participant with a mean BOLD response deviating more than 3 times the median standard deviation was removed as well as their respective twin (number of participants removed = 8). Included in the final analysis was a sample of 56 monozygotic (35 females and 21 males) and 67 dizygotic (39 females and 28 males) twin pairs.

In brief, the phenotypic variance can be decomposed into additive genetic variance (*A*) as genetic effects for a phenotype or trait that add up linearly, common or shared environmental variance (*C*) and unique environmental, or error variance (*E*) (Falconer and Mackay 1996). Using the simplest Falconer’s formula, the *A*, *C*, and *E*-factors can be estimated by contrasting monozygotic-twin pair correlations with dizygotic-twin pair correlations. The *A*-factor can be identified because monozygotic-twins are genetically identical while dizygotic-twins share 50% of their co-segregating alleles on average. Additionally, we assume that a shared environmental contribution (*C*) is equally shared within pairs regardless if they are monozygotic or dizygotic twins. Finally, any variance not attributable to factors shared between twins (*A* and *C*), that is, that make twins in pairs dissimilar, in the model assigned to the *E*-factor. The genetic influence (*h^2^*)*,* is the proportion of a phenotypic variance explained by additive genetic effects, that is, *h^2^* is equal to *A*/(*A* + *C* + *E*). In the present study, we computed heritability using the APACE software package (Accelerated Permutation Inference for the ACE model; Chen et al. 2019). APACE uses a non-iterative linear regression-based method based on squared twin-pair differences, with permutation-based multiple testing correction to control the FWE rate. For the mass-univariate analysis, for each first-level contrast described above, we used the Neurologic Pain Signature as a priori template for regions in which to test for significant differences in genetic influences between twin groups (Wager et al. 2013). The number of permutations was set to 1000 and we used the cluster-based inference in the APACE (accelerated permutation inference for ACE models) software package (Chen et al. 2019) with cluster-forming threshold set to *P* < 0.05 based on the parametric likelihood ratio null-distribution. We additionally computed an estimate of the genetic influence of choice of threshold for the electrical stimulation using the mets package (Scheike et al. 2014; Holst et al. 2016) implemented in R (R Core Team 2017).

### Estimating the Genetic Effect on the Functional Connectome

All individual-level FC matrices (CS+_US_ & US > CS+_no US_) were entered into APACE (Chen et al. 2019) and the genetic influences was computed by fitting the model to each edge in the matrices. This resulted in a 400 by 400 symmetric matrix with *h^2^* estimated for each edge. Subsequently, we used a method based on network-based statistics (Zalesky et al. 2010) to compute a significant cluster or “largest connected component” of the *h^2^* matrix. We ran 1000 iterations and re-computed the 400 × 400 *h^2^* matrix with permuted twin identity. Finally, we computed the largest connected component of our observed *h^2^* matrix and compared with the distribution of randomly generated *h^2^* matrices, determining significance at $\alpha =0.05$. Of note, the network-based statistics approach requires a choice of a threshold for which below all values are set to zero and all values above are set to one. The usage of thresholds that are set too conservatively typically results in network components that are too small to be deemed significant compared with random networks. On the other hand, thresholds that are set too low results in very large network components that are biologically unrealistic. We found that the largest component broke at *h^2^* = 0.328, however we show that there are larger components that are significant by computing components over several thresholds from *h^2^* = 0.25 up to 0.32 in steps of 0.01 (see Supplementary Fig. S5). For interpretability, we chose the component from the largest threshold, denoted the *h^2^*-component (*h^2^* = 0.328) for visualization. To further aid interpretability, we computed the sum of within-network and between-network edges in the *h^2^*-component (Fig. 2*D*). All brain graphs where visualized using BrainNet Viewer (Xia et al. 2013). Node labeling was done with the automated anatomical labeling (AAL; Tzourio-Mazoyer et al. 2002) by taking the coordinates from the Schaefer parcellation (Schaefer et al. 2018) that overlap between the AAL and the *h^2^*-component.

### Notes on the Preregistration

The aim of the current study as stated in the preregistration (https://osf.io/zesw5) was to characterize the genetic influence on FC in pain related brain regions. Our first approach was to use the automated online meta-analysis tool Neurosynth (Yarkoni et al. 2011) to determine the brain regions of interest. We here instead decided to use the Neurologic Pain Signature (Wager et al. 2013), since it is more well-defined and validated. In addition, instead of focusing on the FC between brain regions related to pain, we took a whole-brain approach. This way, we could estimate the genetic influence on functional interactions between nociceptive and non-nociceptive brain regions. We decided furthermore to use weighted functional connectivity (wFC) instead of generalized psychophysiological interactions (gPPI; McLaren et al. 2012) since the former is conceptually simpler, especially since wFC yields undirected graphs, as compared with gPPI that deals with effective connectivity. Future studies can investigate genetic influences on causality in the brain in relation to pain, either through gPPI, granger causality or dynamic causal modeling. Finally, the permutation test based on network-based statistics (Zalesky et al. 2010) was added later, since element-wise (per edge) estimates of genetic influence assumes independence between edges, and would also match the cluster-based statistics from the univariate analysis.

## Results

### Genetic Influence on Brain Activations during Nociceptive Processing

**Figure 1:**
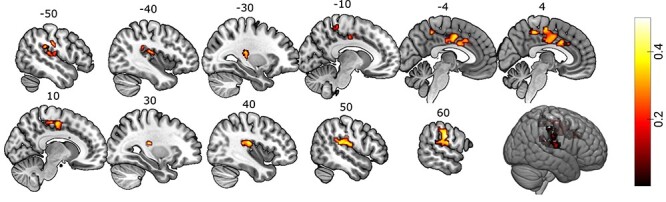
Twin-data brain regions with genetic influences during nociceptive processing. Sagittal view of clusters with significant genetic influence, including the contralateral somatosensory cortex, bilateral dorsal posterior insula, anterior and midcingulate cortex. The threshold was set at *P* < 0.05, FWE-corrected for multiple comparisons at the cluster-level. The heat bar represents *h*^2^ heritability values.

In response to nociceptive stimuli, we detected local increases in BOLD fMRI signals in the bilateral anterior insulae, bilateral posterior insulae, cingulate cortex, thalamus, cerebellum, and the right amygdala (*P*< 0.05, FWE corrected). For a full representation of all regions activated during nociceptive processing see Supplementary SI Appendix, Supplementary Table S1 and Supplementary SI Appendix, Supplementary Figure S2. Estimates of the genetic influence on brain responses during nociceptive processing was constrained to brain regions defined by the Neurologic Pain Signature (Wager et al. 2013). Using permutation tests to assess the degree of genetic influence (*h*^2^ ranging from 0 to 1) on brain activation patterns (Chen et al. 2019), we found significant effects in the right (contralateral) postcentral gyrus (*h^2^* = 0.52), right posterior insula (*h^2^* = 0.50), right superior temporal gyrus (*h^2^* = 0.45), right supramarginal gyrus (*h^2^* = 0.44), left postcentral gyrus (*h^2^* = 0.54), left supramarginal gyrus (*h^2^* = 0.52), left posterior insula (*h^2^* = 0.43), left superior temporal gyrus (*h^2^* = 0.43), left anterior cingulate cortex (*h^2^* = 0.46), right posterior-medial frontal gyrus (*h^2^* = 0.41), and bilateral midcingulate cortex (*h^2^* = 0.40; Fig. 1; see Supplementary Fig. S3 for an unthresholded image of the genetic influence, and see Supplementary Fig. S4 for twin-pair correlations). We note that since CS+_no shock_ was not modeled together with a 3-s period following stimuli offset, this might influence the results. We also performed a symmetric modeling, adding a regressor representing a 3-s period following the offset of CS+ no shock, keeping all other steps the same. Supplementary Figure S5 shows that this step did not affect the group-level GLM results.

### Genetic Influence on FC during Nociceptive Processing

**Figure 2:**
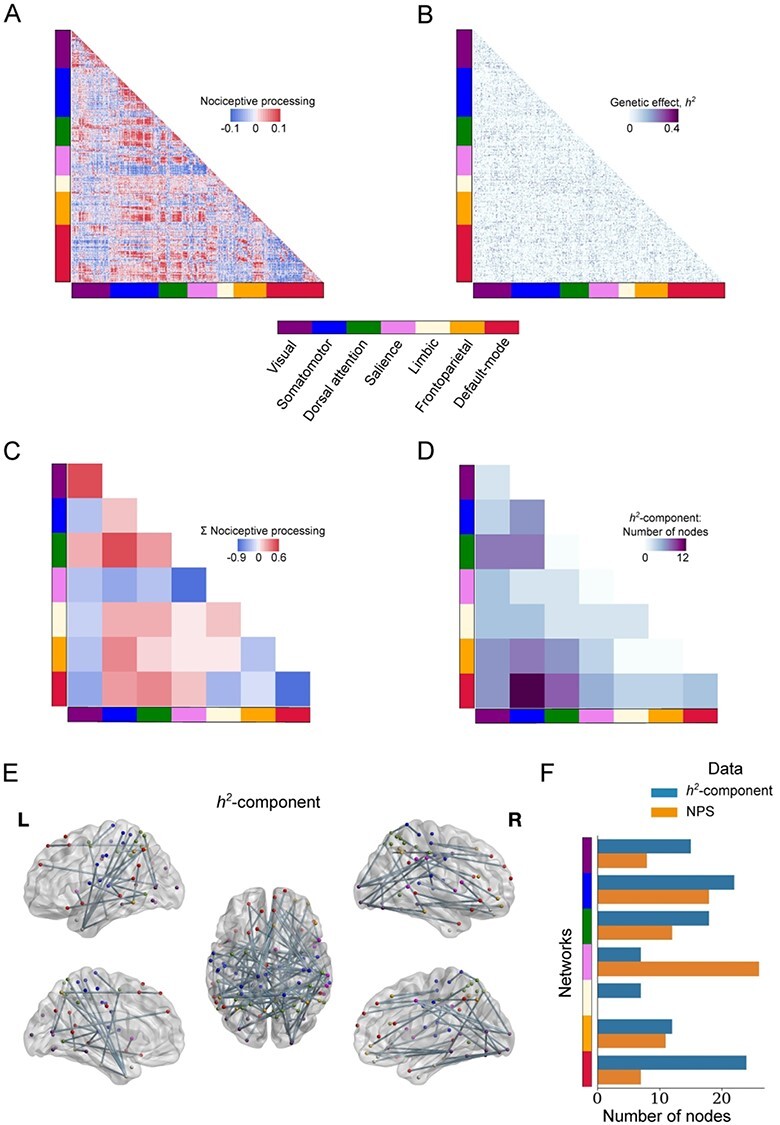
Twin-data FC during nociceptive processing. (*A*) Group-averaged FC during nociceptive processing. Positive values (red) indicate edges with stronger FC during nociceptive processing. (*B*) Unthresholded genetic influence (*h^2^*) for every edge in the FC during nociceptive processing. (*C*) Graphical summary of the FC results in (*A*). The diagonal squares represent the within-network and off-diagonal squares represent the between-network sum of FC during nociceptive processing. Positive values are represented by warm colors. Minimum and maximum values denote the mean ±2 standard deviations. (*D*) The number of edges in the connectivity cluster defined by genetic influence, called the *h^2^*-component, within and between networks. Dark color denotes higher number of edges. The largest number of edges was found between the somatomotor and default-mode network. (*E*) Brain graph representing the *h^2^*-component from (*D*). The edges comprise a *h^2^*-component that represents significant genetic influences on nociceptive processing (*P* < 0.05, corrected, *h^2^* threshold = 0.328). (*F*) The number of nodes in the parcellation scheme that overlap with the *h^2^*-component (blue) (defined with a threshold of *h^2^* = 0.328) or the Neurologic Pain Signature (orange).

During nociceptive processing we observed increases in FC between several brain networks, including the somatomotor and dorsal attention networks (Fig. 2*A* and *C*). The FC within the default-mode-network decreased during nociceptive processing and increased within the visual network. To estimate the genetic influence on FC, we used a permutation test based on network-based statistics (Zalesky et al. 2010). This approach allowed us to identify a cluster of connections from the full *h^2^*-matrix (Fig. 2*B*), where each connection represents the genetic influence on FC (Fig. 2*D*–*F*; thresholded at *P* < 0.05, corrected using 1000 permutations). The most conservative threshold where a significant cluster of connections could be determined (*h^2^*-component) was *h^2^* = 0.328 (see Supplementary Fig. S6 for a comparison to other thresholds). The edges of the *h^2^*-component linked together brain regions located within as well as outside the Neurologic Pain Signature (Fig. 2*F*). Nodes within the *h^2^*-component were spatially situated in the dorsal posterior insula, anterior-, mid- and posterior cingulate cortex, precuneus, and orbitofrontal cortex.

### Genetic Influence on Behavioral Sensitivity to Electrical Stimuli

There was a significant genetic influence on nociceptive thresholds, based on perception-matched aversive electrical stimuli (*P* < 0.0001, *h^2^* = 0.18, on choice of threshold). Estimates of the between-twin correlation of nociceptive thresholds for monozygotic twins was higher (*r* = 0.18 and 95% CI = [−0.02–0.38]) than the between-twin correlation for dizygotic twins (*r* = 0.09 and 95% CI = [−0.01–0.19]).

## Discussion

There is high variability in the way humans respond to nociceptive stimuli and express pain, yet there is little knowledge about the contributions of nature versus nurture to this variation. In this study, we used a twin-study approach to determine the magnitude and spatial representation of genetic influences on brain circuits involved in nociceptive processing. We found significant genetic influence on activity in brain regions typically activated by nociceptive processing (Fig. 1 and Table 1). Interestingly, genetic influence on nociceptive FC was not restricted to these areas but also included regions across the brain (Fig. 2*D*–*F*).

**Table 1:** Genetic influence, *h^2^* during nociceptive processing (*P* < 0.05, FWE corrected)

	MNI				
Area of local maximum	*h* ^2^	*X*	*Y*	*Z*	Voxels in cluster
R Postcentral	0.52	60	−16	30	876
R Insula	0.5	30	−20	18	
R Superior temporal gyrus	0.45	54	−30	18	
R Supramarginal gyrus	0.44	56	−24	18	
L Supramarginal gyrus	0.54	−65	−20	28	627
L Postcentral gyrus	0.52	−62	−22	32	
L Insula	0.43	−36	−20	18	
L Superior temporal gyrus	0.43	−64	−30	22	
L Anterior cingulate	0.46	0	20	28	620
R Posterior-medical format	0.41	2	−14	58	
R Midcingulate	0.40	8	−14	40	
L Midcingulate	0.40	−2	−4	40	

*Notes:* R = right hemisphere, L = left hemisphere.

Nociceptive responses in bilateral dorsal posterior insula and mid/anterior cingulate cortex were influenced by genetics (Fig 1 and Table 1), even if the cluster on the contralateral insular side was more pronounced. Previous studies have suggested that the dorsal posterior insula may be of importance for nociceptive processing (Segerdahl et al. 2015). It is a primary projection point from the ventral medial nucleus of the thalamus and constitutes a core pathway for nociception in all primates (Craig 2003). This thalamocortical pathway is believed to provide a sensory reflection of the condition of the body, and thereby has great evolutionary value (Craig 2003). This is corroborated by fMRI data from new-born babies as it reveals a large overlap between nociceptive processing in adults and infants, including the thalamus, insula and mid/anterior cingulate cortex (Goksan et al. 2015). This network could be considered as potential targets in studies searching for markers of chronic pain and novel treatment, especially for conditions with known familial risk. Genetic variability is likely to be involved in the mechanisms underlying some of our most common pain conditions (Parisien et al. 2017) but the mediating mechanisms are poorly understood. The results presented here demonstrate that nociceptive processing is significantly influenced by genetics and is likely to mediate the different nociceptive processing seen in individuals with chronic pain (Jensen et al. 2009; Hashmi et al. 2013).

Regarding the FC results, we observed that the nodes within the so-called h^2^-component (connectivity influenced by genetics) were members of several different networks, most notably, the somatomotor, default-mode, and dorsal attention networks (Fig. 2*D*–*F*). This indicates that genetic influence on FC during nociceptive processing encompasses both sensory and affective-cognitive processes. Since nociception is shaped by interactions between sensory, cognitive, and affective processes there is indeed a possibility that some aspect of all these components is heritable. The brainwide pattern of connectivity might reflect the integration of functionally specialized subsystems such as attention and somatosensory processing. We observed that the largest number of connections in the *h*^2^-component was found between the default-mode and somatomotor networks (Fig. 2*D*) even though FC between the 2 was not the strongest (Fig. 2*A* and *C*). Several studies show evidence for the involvement of default-mode and somatomotor network in pain processing (Kong et al. 2010; Kucyi et al. 2013; Goffaux et al. 2014; Kim et al. 2019; Perini et al. 2020). In general, genetic influence on the integration between multiple networks may support the multifaceted experience of nociception and pain. For example, genetic influence on the level of integration between attention and nociception could give rise to individual differences in pain sensitivity.

Here, we isolated the genetic contribution to task-evoked FC. Yet, several findings show great similarity between task-evoked and resting-state FC (Fox and Raichle 2007; Cole et al. 2014). Such similarities, however, should not be transferred by analogy to a comparison between resting-state and pain-evoked FC. Even comparing non-painful and painful stimuli shows marked differences whereby the former resembles a network formation akin to resting-state (Zheng et al. 2020). The cluster of edges identified in the present study captures variance associated with additive genetics supporting the search for a genetically informed neural pain signature (Davis et al. 2020). Future studies should compare resting-state and task-evoked FC and estimate the extent of their shared genetics and the neural targets of their shared and non-shared genes.

The role of genetics for the variability in nociceptive processing, is likely expressed at all levels of the neural axis, for example a recent study suggested a link between a Neanderthal gene (*SCN9A*), and the initiation of nociceptive signaling in the periphery (Zeberg et al. 2020). In the brain, however, the nociceptive signal is represented in several brain regions, and thus far it has been difficult to determine which aspects of nociception are heritable and which ones are shaped by life experience. The data in the present study provides the first genetically informed nociceptive signature that distinguishes between heritable and acquired nociceptive responses in the brain. There is currently a need for better characterization of the biological and genetic foundations of the neural representation of pain. One review and a recent consensus paper by leading pain clinicians and scientists (Tracey et al. 2019; Davis et al. 2020) explicitly ask for pain biomarkers—verifiable in preclinical models and patients. Stratification biomarkers may increase the probability of success in pharmacological clinical trials by as much as 21% in phase III clinical trials in all disease areas (Davis et al. 2020). Our results may help determine if clinical pain is manifested in genetically inferred nociceptive regions, and hopefully lead to beneficial sub-grouping and patient stratification.

We note that the experiment also included a fear conditioning task, which entails a risk that our findings are confounded by cognitive and affective processes related to learning and anxiety. Related to this, there are other psychological factors that are heritable, for example anxiety, that could influence nociceptive processing. We can, therefore, not exclude the contribution of closely related heritable factors to our findings. For example, genetic factors could influence anxiety that in turn influence nociceptive processing. On the one hand, our analytical approach aimed to isolate the effects of the nociceptive stimulus itself and hopefully minimized any brain activations related to the fear learning component of the experimental paradigm. On the other hand, there is an inherent affective component of nociception and it will thus be difficult to remove all fear-related brain activations as they may also be present during the nociceptive stimulation modeled in our analysis. An experimental design that controls for related features, such as salience, could more clearly probe the genetic influence of network-level processes underlying nociception and pain. As we have noted, however, pain and nociception are composed of multiple features and it may be difficult to part these in fMRI.

Besides the difficulty of dissociating nociception, or pain, from other features of the brain, there are some limitations that need to be considered in interpreting our results. We examined the genetic influence on nociceptive processing and not subjective pain. Although the nociceptive stimuli in our study represent aversive events in the sensory domain (Lee et al. 2020), participants did not provide subjective ratings of pain. This would have allowed a clearer relationship between genetic influences on brain activation and FC with the subjective pain experience. Further, this study examined only one nociceptive modality—electrical stimulation. Even if our findings elucidate heritable neural mechanisms that overlap with findings among patients with clinical pain they may not generalize to a clinical context. If we had used other nociceptive stimuli that stimulate deeper tissues, and provide C-fiber mediated activations, it would have made a stronger case for a possible clinical translation. Finally, the sample size is relatively small and may be underpowered to detect some effects. With our sample size, reaching 80% power (*P*_α_ = 0.05) requires the true effect of additive genetics to be 0.5. These calculations (Visscher 2004; Visscher et al. 2008) are, however, based on a generic tool for twin studies and may not be comparable to the statistics of neuroimaging.

To summarize, our twin-design elucidates specific aspects of nociceptive processing in the brain that are under genetic influence. Also, the genetic influence on FC during nociceptive processing is not limited to core nociceptive brain regions, such as the dorsal posterior insula and somatosensory areas, but also involves cognitive and affective brain circuitry. Recent efforts to characterize the association between functional brain networks and gene expression (Richiardi et al. 2015) point to the importance of factoring in genetics in the mapping of human brain function. A genetically informed model of nociceptive processing in the brain is likely to complement recent studies suggesting a clinically relevant signature of pain (Lee et al. 2021) and provide insights into clinical pain conditions.

## Authors’ Contributions

J.R. and F.Å. designed the experiment. G.K. and J.R. performed the experiments. G.K. and K.J. wrote the first draft. All authors substantially revised the manuscript. X.C. and T.N. contributed with the software. G.K. analyzed the data and G.K., X.C., W.H., T.N., I.T., P.F., F.Å., and K.J. contributed to interpretation of results. All authors have read and approved the manuscript.

## Notes

*Conflict of Interests:* H. Larsson has served as a speaker for EvolanPharma and Shire/Takeda and has received research grants from Shire/Takeda; all outside the submitted work. All other authors declare no competing interests.

## Funding

Swedish Research Council (grant 2014-01160, 2018-01322 to F.A.). A generous donation from the Leif Lundblad family made this study possible.

## Data and Materials Availability

The data generated during the current study are available at osf.io with DOI 10.17605/OSF.IO/UWYEV. Further data and all the code used to produce figures and results are available at https://github.com/granitz/twin_pain.

## Supplementary Material

supplementary_files_bhab206
