# Supplementary material for: Genetic Influence on Nociceptive Processing in the Human Brain—A Twin Study
Source: Cereb Cortex. 2021 Jul 21;32(2):266–74. doi: 10.1093/cercor/bhab206 (PMC8754385; doi:10.1093/cercor/bhab206)
Supplement: supplementary_files_bhab206 [file supplementary_files_bhab206.zip › Revised Supplemental_final.docx]

Supplementary Material

**Genetic influence on nociceptive processing in the human brain – a twin study**

Gránit Kastrati^1,2^, Jörgen Rosén^2^, William H. Thompson^1^, Xu Chen^3^, Henrik Larsson^4^, Thomas E. Nichols^5^, Irene Tracey^6^, Peter Fransson^1^, Fredrik Åhs^2^ & Karin B. Jensen^1^*

^1^Department of Clinical Neuroscience, Karolinska Institutet, Stockholm, Sweden.

^2^ Department of Psychology and Social Work, Mid Sweden University, Östersund, Sweden.

^3^ Department of Biomedical Data Sciences, Leiden University Medical Center, the Netherlands.

^4^ Department of Medical Sciences, Örebro University, Örebro, Sweden.

^5^Oxford Big Data Institute, Li Ka Shing Centre for Health Information and Discovery, Nuffield Department of Population Health, University of Oxford, Oxford, U.K.

^6^ Wellcome Centre for Integrative Neuroimaging, Nuffield Department of Clinical Neurosciences, University of Oxford, Oxford, U.K.

*Correspondence to: Karin.Jensen@ki.se

**Content:**

Figs. S1 to S6

Table S1

Captions for Data S1

Captions for Data S2


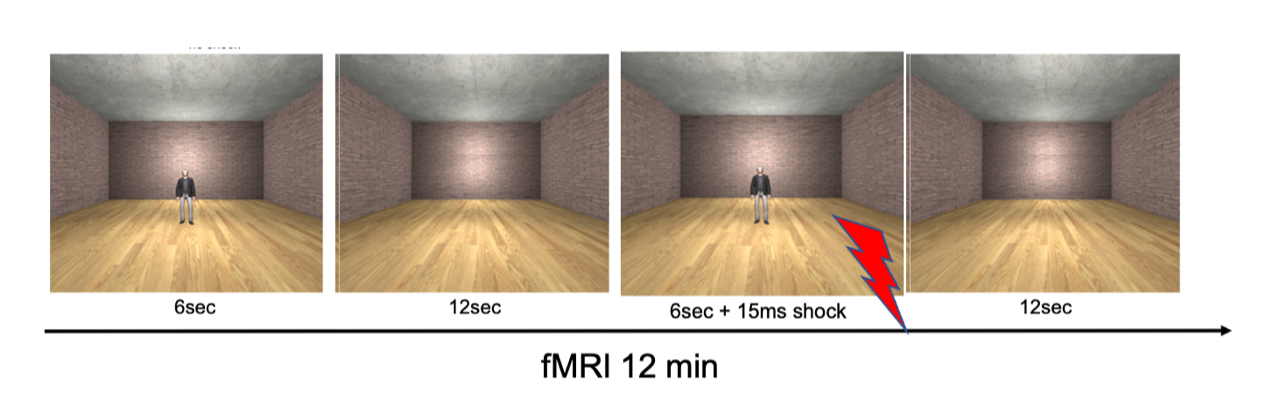


Fig. S1. Experimental design. In the original experiment, two virtual characters served as conditioned stimuli (CS). One of the virtual characters served as aversive cue (CS+) and predicted the electrical shock and the other cue served as control (CS-) and was never coupled with the shock. Each CS appeared for 6s with an inter-stimulus interval of 8-12s. Prior to the experiment, participants were not told which character would be associated with electrical shocks. During conditioning, each CS type was displayed 16 times. Eight of the CS+ presentations co-terminated with a brief (15ms) electrical shock, and eight presentations of the same visual cue was not followed by a shock. In the present study, only the CS+ cue was included in the analysis to compare brain activations for CS+ followed by a shock versus CS+ not followed by a shock.

Fig. S2. Brain responses during nociceptive processing. (A) Images represent whole-brain activity to nociceptive stimuli (p<0.05, *FWE* corrected). (B) Same as in (A) but here limited to regions within the borders defined by the Neurologic Pain Signature.

Fig. S3. Unthresholded genetic influence, *h^2^*. Genetic influence was estimated per voxel on individual level contrast images masked with the Neurologic Pain Signature.

Fig. S4. Unthresholded whole-brain between twin-pair correlations for (A) identical or monozygotic and (B) fraternal or dizygotic twin-pairs.

**Fig. S5**. Second-level GLM performed with symmetric modelling. A new regressor was included with a 3s duration following CS+ no shock. The figure shows the original group-level result (**A**), the new (**B**) and the two combined (**C**). Results are FWE corrected results at p = 0.05.

Fig. S6. The *h^2^*-components for different thresholds of *h^2^*. With a lower threshold value, larger but weaker connected components are identified.

Table S1.

Brain areas activated during nociceptive processing (*P <0.05, family-wise error corrected*). R = right hemisphere, L = left hemisphere. Note that SPM Anatomy identified only one cluster.

Data S1. (separate file)

Niftii files corresponding to unthresholded a^2^, c^2^ and e^2^ are available at https://github.com/granitz/twin_pain.

Data S2. (separate file)

Niftii files corresponding to the original group-level GLM and a new image that was modelled symmetrically, with a regressor modelling the time (3s) following CS+_no shock_, is available at https://github.com/granitz/twin_pain.
